# Getting Out of the Classroom and Into Nature: A Systematic Review of Nature-Specific Outdoor Learning on School Children's Learning and Development

**DOI:** 10.3389/fpubh.2022.877058

**Published:** 2022-05-16

**Authors:** Jeff Mann, Tonia Gray, Son Truong, Eric Brymer, Rowena Passy, Susanna Ho, Pasi Sahlberg, Kumara Ward, Peter Bentsen, Christina Curry, Rachel Cowper

**Affiliations:** ^1^School of Education, Western Sydney University, Kingswood, NSW, Australia; ^2^School of Health and Human Performance, Dalhousie University, Halifax, NS, Canada; ^3^Discipline of Psychology, Southern Cross University, Bilinga, QLD, Australia; ^4^Plymouth Institute of Education, University of Plymouth, Plymouth, United Kingdom; ^5^SR Nathan School of Human Development, Singapore University of Social Sciences, Singapore, Singapore; ^6^Faculty of Education, Southern Cross University, Lismore, NSW, Australia; ^7^School of Education and Social Work, University of Dundee, Dundee, United Kingdom; ^8^Department of Geosciences and Natural Resource Management, University of Copenhagen, Copenhagen, Denmark; ^9^Center for Clinical Research and Prevention, Copenhagen University Hospital, Frederiksberg, Denmark; ^10^Thrive Outdoors, Inspiring Scotland, Edinburgh, United Kingdom

**Keywords:** academic improvement, education, learning outside the classroom, nature-specific, outdoor learning, pro-environmental behavior, socio-emotional, wellbeing

## Abstract

**Background:**

The value of natural environments for developing children's self-identity and social skills has been known for some time, and more recently the potential of nature-specific (i.e., excluding built environments) outdoor learning for achieving academic outcomes has been explored. Connecting children with natural spaces has been shown to benefit their physical and mental health; however, the utility of nature-specific outdoor environments as a setting for curricular and non-curricular learning has yet to be clearly established. Our aim was to undertake a narrative synthesis of international evidence of nature-specific outdoor learning and its benefits for personal and social development, wellbeing and academic progress.

**Methods:**

This systematic review searched publications between 2000 and 2020 in nine academic databases for evidence of socio-emotional and academic benefits of nature-specific outdoor learning in school-aged educational settings, using concise search criteria registered with PROSPERO. The total search results of 17,886 records were initially screened by title, and then two reviewers made blind reviews of the title and abstract of 1,019 records.

**Results:**

147 original research studies meeting the criteria were identified. Learning settings ranged across outdoor adventure education, school gardens, field trips, and traditional school subjects taught in natural environments. Study characteristics were summarized, and risk-of-bias tools assessed quality of research as generally moderate, although with a wide range. The reported benefits of learning in natural outdoor settings include: increased student engagement and ownership of their learning, some evidence of academic improvement, development of social and collaborative skills, and improved self-concept factors.

**Conclusions:**

Nature-specific outdoor learning has measurable socio-emotional, academic and wellbeing benefits, and should be incorporated into every child's school experience with reference to their local context. Teacher pre-service and in-service education needs to include a focus on how natural settings can be used effectively for learning. Further research is needed to clarify the conditions under which specific forms of outdoor learning are most efficacious for various target outcomes. It is recommended that future studies measuring outdoor learning adopt established methodologies to improve the quality of research in this field.

**Systematic Review Registration:**

https://www.crd.york.ac.uk/prospero/display_record.php?RecordID=153171.

## Introduction

An international “renaissance of interest” (1) has emerged in learning outside the classroom in recent years, as the shortcomings of a test-dominated model of education have become apparent. Sahlberg (2) for instance, commented that this narrowly focused test-oriented model of education reduces the range of learning activity for students, and has the effect that “when educational performance is determined by students' test scores in reading, mathematics, and science, it reduces focus on whole-child development due to decreasing time for arts, music, drama, and sports” (p. 138). At the same time, there is an increasing body of research that demonstrates how students can benefit from learning outside, not only in terms of the curriculum, but also with respect to social, personal, and physical development (3, 4). UNICEF's report on the factors that shape child well-being in affluent countries lists “good mental well-being” as a “key aspect of quality of life”, and notes that “ore time playing outside is linked to much higher levels of happiness” (5). There is strong resonance with a frequently held principle in many Western, and especially Scandinavian, countries, that nature must play an integral role in childhood education (6–9).

This paper presents an examination of the literature on nature-specific learning outside the classroom (NSLOtC) and the types of benefits and/or impacts such opportunities have on children. Our scoping paper (10) used the term “outdoor learning”, however we have chosen the more precise term of NSLOtC. Following Atencio et al. (11), NSLOtC includes practical and experiential learning activities conducted outside in school grounds and other locations such as parks, forests, residential camps/centers or on expeditions. Activities can be curricular or non-curricular, focus on different areas of cognitive, social, emotional and moral development, and be related to indoor learning. This type of learning may take place during curricular time or outside school hours. It is narrower in scope than generalized learning outside the classroom (LOtC), which includes off-campus learning in natural or built environments and/or visits to sites such as museums, galleries and/or historical monuments (12). Whilst there have been systematic reviews of research on exposure to natural environments without regard to learning, and on LOtC irrespective of whether the setting is natural or built, this paper draws together research on various forms of learning in natural environments, across formal and informal curricula, spanning a range of school student age groups.

### Benefits of Being in Nature

A mounting body of research clearly indicates that spending time in natural settings which have not been “hardened” or “improved” is good for human and planetary wellbeing (8, 13–15). However, people in industrialized and urban settings are spending less time outdoors (16). In particular, children are moving indoors at a vital time in their growth and development when the evidence-base suggests they would benefit from time outdoors (7, 17). This reduction of interaction with nature is reflected in the experiences of young people, and is becoming increasingly apparent in educational settings worldwide (8, 18). Ensuring children have adequate access to nature is of critical importance for their health and wellbeing (13, 15, 19). However, while there has been an international recognition that schools are vital for enhancing wellbeing in young people (20) and for developing pro-environment attitudes and behaviors (21), most schooling is guided by the concept of indoor learning and four-walled classrooms.

### Nature, Wellbeing and Young People

As noted above, student wellbeing has become an important aspect of education, and often refers to overall development and quality of life. While there is no one commonly agreed definition of wellbeing, descriptions often focus on psychological notions such as positive mental health, a sense of purpose and belonging, high life satisfaction and the ability to manage stress and life challenges (22). General agreement, however, exists about the minimum conditions of wellbeing, which include the presence of positive emotions, life satisfaction, fulfillment and positive functioning (23). There have been two main perspectives on wellbeing namely, hedonistic and eudemonic wellbeing. The former relates to finding pleasure and avoiding pain, whilst the latter refers to meaning and purpose. Though still in the early development of hedonistic psychology, these were considered to be distinct entities, and recent literature on wellbeing has promoted a more holistic approach incorporating both approaches (22). Studies examining wellbeing in young people have also included social (e.g., relationships), environmental (e.g., integration with nature) and physical dimensions of wellbeing, as well as the importance of play, learning, a sense of belonging and life satisfaction to the perception of wellbeing (24, 25). Wellbeing is a complex, multi-dimensional construct, which cannot be measured by using a single indicator in any given context (26). Research that measures student wellbeing needs to be based on the multi-dimensional nature of wellbeing, such as the right of children to happiness, and the importance of their ability to enhance their wellbeing in the present and in the future (27).

The multifaceted nature of wellbeing means that various domains can differently affect students' life satisfaction and wellbeing, with each domain operating as a condition for and a consequence of the other domains (28). It can therefore be argued that the physical environment in which these domains operate has a considerable impact. Research shows that access to open and natural spaces supports improved physical health, with time outside in nature also having beneficial effects on cognitive and mental health (29).

Several systematic reviews have assessed wellbeing outcomes from interactions in natural environments for various populations. For example, Gill (30) reviewed 71 studies involving primary aged children's experience of nature, and found benefits to mental health, emotional regulation and environmental knowledge and attitudes. Holland et al. (31) concluded that “wildland” recreation resulted in psychological, social, and educational benefits, across 235 studies with mostly adult participants. Tillman et al. (32) similarly explored the effect of nature on mental health specifically in participants aged 0–18 years old. An experience of nature positively influenced mental health across the 35 studies, however only 15 of these involved active engagement with nature rather than passive exposure.

Three systematic reviews shared the focus of this review on educational experiences in natural settings. Becker et al. (3) reviewed 13 studies with regular weekly or fortnightly classes in the outdoors, and found emerging evidence for social, academic, physical and psychological change. Mygind et al. (19) examined immersive nature learning experiences, most of which were secondary school aged participants in expedition and residential adventure programs. They identified 84 studies, and further analyzed 36 quantitative studies, finding immediate benefits to a range of mental, physical and social health outcomes. Finally, Miller et al. (33) investigated nature-based learning in primary aged children, and observed positive evidence for social and educational development, and emerging evidence for engagement, mental health and wellbeing. Collectively, these reviews support the value of nature immersion for people of all ages, and various educational benefits of nature-specific learning for students from particular age groups and outdoor learning settings. This review aims to build on these previous efforts in order to clarify the socio-emotional and academic benefits of NSLOtC for all school aged children across a range of outdoor contexts.

In summary, engaging with nature is important for the health and wellbeing of young people, and providing opportunities that best leverage this relationship is likely to have the greatest impact. NSLOtC could be a vital medium for developing wellbeing and understanding how best to facilitate these experiences is essential to ensure effective learning design.

### The Emergence of Nature-Specific Learning Outside the Classroom

Most government policymakers, curriculum designers and school leaders recognize the importance of psycho-social and wellbeing factors for students' development as well as academic outcomes (34). An emerging interest in NSLOtC has led many schools and individual teachers to consider how learning in nature can realize these potential benefits. Indeed, across the world, NSLOtC is becoming increasingly common. For example: the “udeskole” Scandinavian philosophy of curriculum learning in local outdoor settings (35), the outdoor adventure education movement pioneered by Outward Bound (36), active learning in Scotland (37), and experiential learning in school gardens (38). However, despite this growing interest, there is a dearth of guidance as to which NSLOtC approaches are appropriate in various contexts, and what specific outcomes they might achieve and for whom (33). This limits the potential leveraging of NSLOtC settings to improve learning and health and wellbeing outcomes for children and young people, potentially leading to inefficient and ill–targeted investment decisions.

This review provides clarification of the benefits of NSLOtC experiences of school children across all ages, incorporating a range of curricular and non-curricular learning contexts. The aim is to provide a resource for decision-makers in government, university pre-service teacher education, school districts and other interested groups by outlining: possible interventions; the potential development, wellbeing and academic learning outcomes of NSLOtC; and, the target beneficiaries.

## Methods

**Figure 1:**
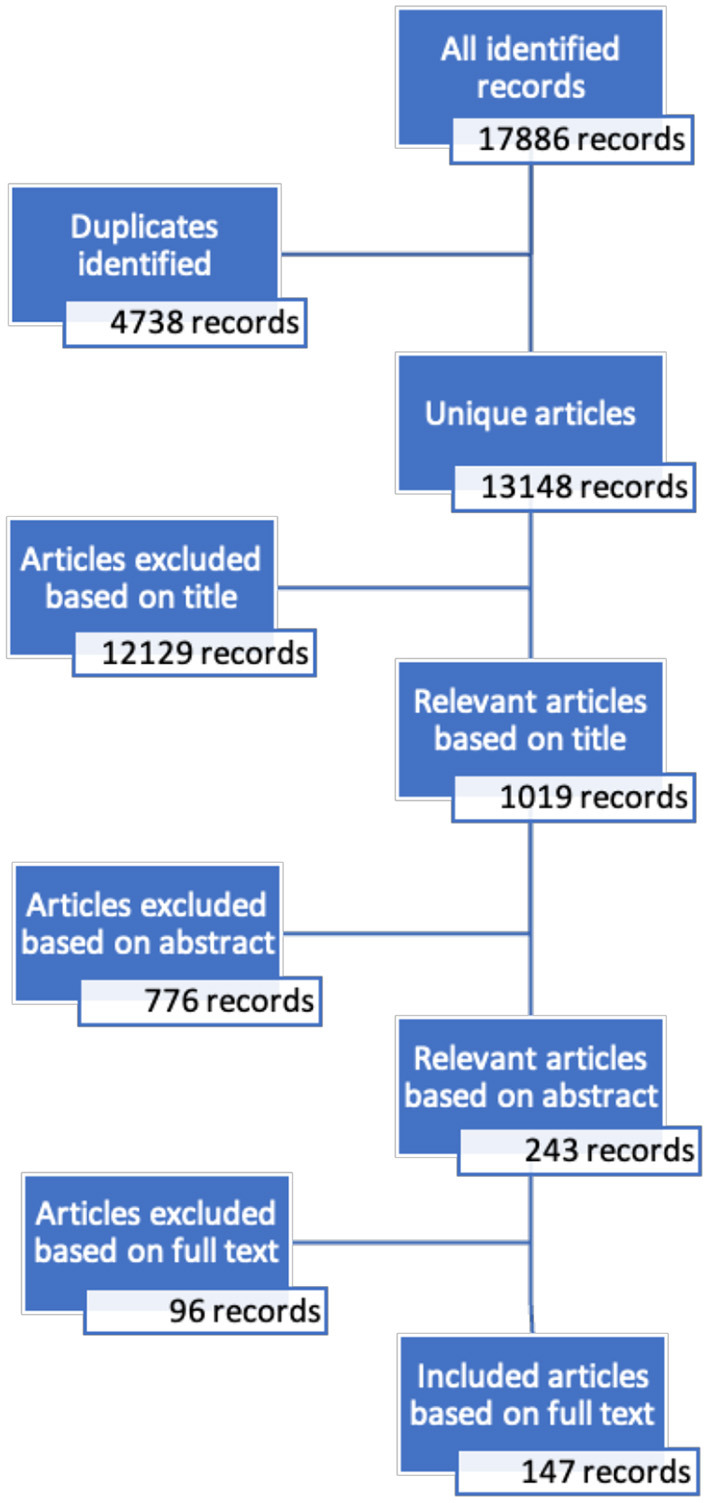
Screening process.

The search procedure was registered with the International Prospective Register of Systematic Reviews (PROSPERO) Number CRD42020153171. Figure 1 describes the screening process. Nine databases (ERIC, ProQuest, PSYCInfo, PubMed, Sage, Scopus, Taylor and Francis, Web of Science, Wiley) were accessed by the international review team, yielding 17,886 hits with a keyword in the each of the three categories (education outside the classroom, learning, and wellbeing) and published between 2000 and 2020, outlined in our protocol paper (10). Duplicates were removed in Endnote, and 13,148 unique records were copied to an online screening tool, Rayyan (https://www.rayyan.ai/).

A detailed description of inclusion and exclusion criteria is covered in Mann et al. (10). The criteria for inclusion in this systematic review were:

- School-aged participants (Kindergarten to Year 12).
- Learning substantively based in a natural outdoor setting.
- Regular sessions, or a multi-day residential program.
- A measurable learning outcome (academic, wellbeing or socio-emotional).
- Original research published in English.

The principal reviewer screened records by title to remove studies that clearly did not meet the inclusion criteria, yielding a total of 1,019 records. Two reviewers then read the title and abstract and made blind inclusion decisions, with any conflicts resolved collaboratively between them. This further reduced the list to 243 records. Lastly, the full text of each article was read and screened by one member of the review team, with conflicts resolved by the principal reviewer, resulting in a final total of 147 included records.

Reviewers recorded the study characteristics of each included article and used established checklist tools to rate the qualitative and/or quantitative research quality in the study. Quantitative studies were assessed using the CCEERC tool (39), which rates factors including: participant selection and sample size, operationalisation of concepts, appropriateness of statistical techniques and ethical standards. Qualitative studies were similarly assessed with the JBI Checklist (40), which incorporates: congruity between the research methodology with methods, analysis and interpretation of results, representation of the participants' voices, influence of the researcher, and ethics standards. These checklist tools require a rating of +1, 0 or −1 for each of 11 and 10 questions, respectively, and were adjusted to produce a score between −10 and +10. The full list of rating items and checklist references are included in Mann et al. (10).

A preliminary survey of the measured outcomes was conducted before identifying outcome categories. The three broad areas of learning defined in the literature search (socio-emotional, academic and wellbeing) provided a lens for determining these categories.

## Results

This systematic review of NSLOtC research began with a broad reach and included a large number of primary studies, compared with similar systematic reviews [e.g., 3, 19, 33]. A total of 147 studies were included in the final review.

### Study Characteristics

**Figure 2:**
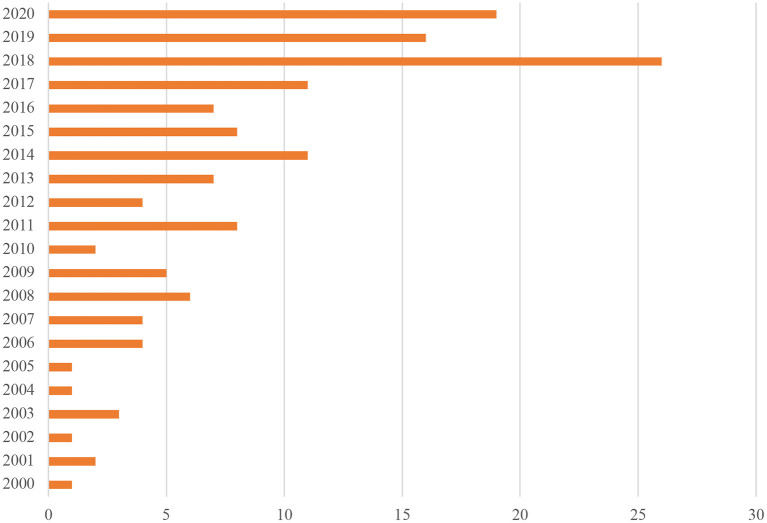
Published studies by year.

The dataset included research conducted across 20 countries, with most studies conducted in the United States of America (54). Multiple studies were also conducted in the United Kingdom (27), Australia (15), Canada (8), Denmark (6), New Zealand (6), Spain (4), South Africa (4), Sweden (3), Germany (2), Singapore (2) and Turkey (2). Frequency of research generally increased over time, with the most studies published in 2018 (see Figure 2).

**Figure 3:**
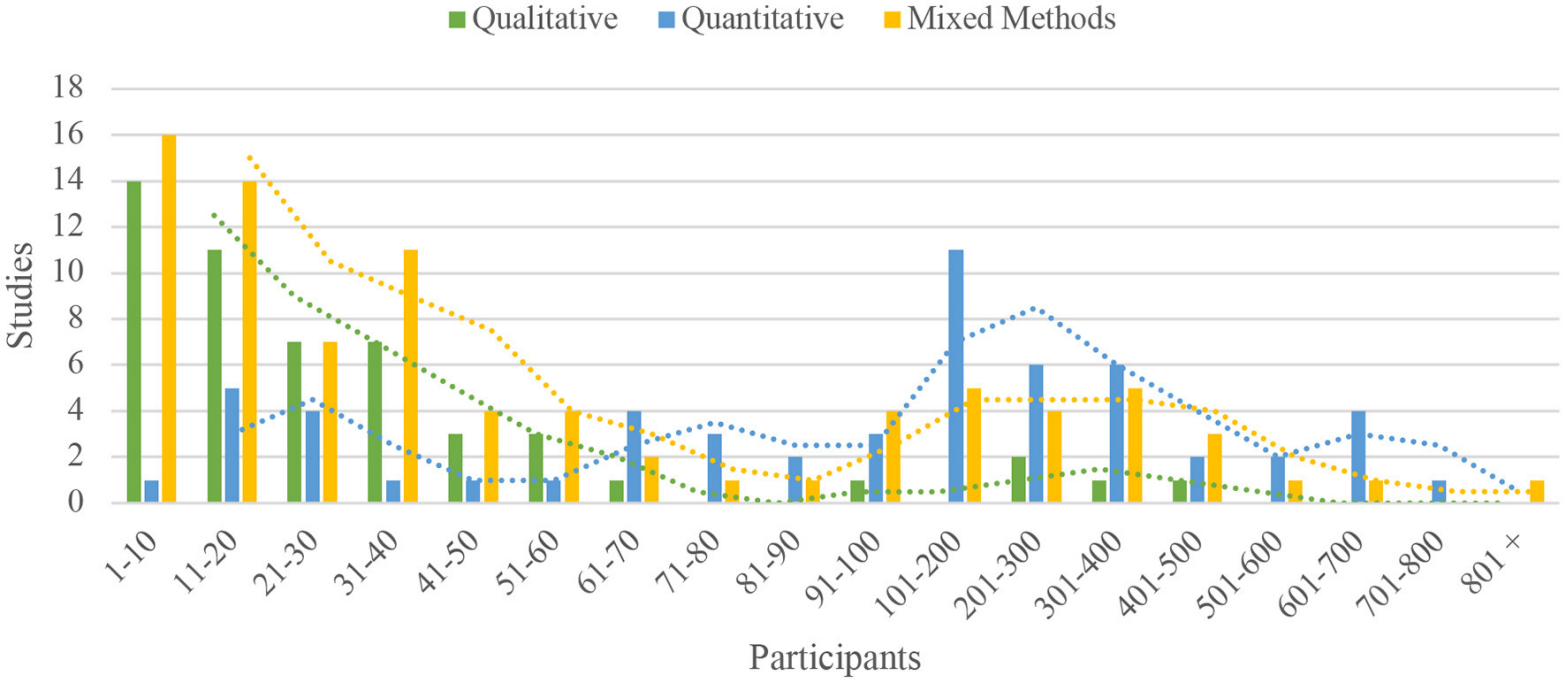
Participant number by research design type (grouped by tens then hundreds).

The number of participants in each study varied greatly, from qualitative (*M* = 47, SD = 88) to quantitative (*M* = 208, SD = 200) and mixed method (*M* = 191, SD = 234) research designs. Figure 3 provides a graphical representation of participant numbers, grouped by tens then hundreds. The majority of studies (60%) were conducted with secondary (Year 7–12) school aged participants, while 36% involved primary (Year 3–6) and 4% were carried out with infants' (Kindergarten—Year 2) school aged children (see Table 1). Almost all studies (127) had mixed gender participants, with only 11 female-only studies, 5 studies in male-only settings, and 8 studies where participant gender was not specified.

**Table 1:** Outdoor learning contexts by participant age.

	**Infants**	**Primary**	**Secondary**	**Total**
Adventure therapy (i.e., using adventure as a context for treatment)			4	4
Adventure education (e.g., canoeing expedition)		2	35	37
Regular field trips (e.g., environmental projects)	1	6	4	11
Curricular lessons in local outdoors (e.g., writing poetry in the forest)		23	6	29
Residential camp (e.g., science camp)		15	23	38
School grounds (e.g., playground)	1	1	1	3
School gardens (located in the school, or in the local community)	2	16	8	26
Other	1	2	5	8
Total	5	65	86	156

*Studies which had multiple ages were counted for each category*.

Categories for outdoor learning contexts were formed after an initial survey of the dataset, and Table 1 shows that the most common contexts were adventure education (25%), residential camps (22%), curricular lessons conducted in the outdoors (20%) and school gardens (16%). Regular field trips (7%), adventure therapy (3%) and school grounds (2%) were less common contexts for research. Adventure education studies were conducted almost exclusively with secondary students, which may be reflective of practice in this field. In contrast, curricular lessons in the outdoors and school garden learning were studied predominantly with primary students. Research into residential camps was well represented across both age groups, however more studies were conducted with secondary students.

Learning outcome categories were similarly developed after a preliminary inspection of the data, informed by the three literature search areas (academic, socio-emotional, wellbeing). The most common reported outcomes were so called “soft skills”, relating to a student's developing understanding of their self-concept and intrapersonal skills (e.g., self-confidence, resilience) and their social and interpersonal skills (e.g., communication, teamwork). Wellbeing (e.g., mood, positive self-concept, sense of calmness) was a pre-determined area of interest, and was combined with mental health (e.g., emotional health functioning, self-determination factors) as these outcomes were intertwined. As well as summative academic progress (e.g., reading level, mathematics development), a number of studies focused on formative academic learning skills (e.g., planning, critical thinking) and engagement with learning (e.g., motivation to be at school, classroom engagement), and a few studies measured school attendance after participation in NSLOtC.

Environmental knowledge and attitudes (e.g., knowledge about and interest in animals, environmental stewardship) were not anticipated to be a discrete category from general academic outcomes, however a significant proportion of studies focused specifically in this area which justified its own outcome category. A recent report published by the Child and Nature Network (41) examined the growing body of evidence that shows how interactions with nature influence positive environmental behaviors. For example, stewardship and positive conservation behavior can be promoted by spending time in nature, having role models in care for nature, and positive experiences in nature. In order to support children and young people in becoming agents of change, Chawla (42) proposed place-based learning experiences and opportunities for agency and authentic participation in civic decision-making and actions related to the local environment. This is echoed by others who argue the need to go beyond cognitively-oriented engagement with the natural world to include the affective domain and opportunities for genuine agency (41, 43, 44). Prince (45) argued that outdoor learning facilitates pro-environmental behavior in children through role modeling, the provision of mentors and modeling actual practice in context. Echoing this, Brymer and Davids (46) highlighted the need to ensure that outdoor experiences designed to enhance pro-environmental behaviors were directly related to the everyday context of the learner. This implies we need to reconsider the role of nature experiences in education and to recognize that successful implementation requires experiential approaches which engage all domains of learning in context.

Personal and social development outcomes were measured most frequently (see Table 2), including: intrapersonal (24%) and interpersonal (19%) development, along with the related factors of mental health and wellbeing (14%). Environmental knowledge and attitudes were assessed in 17% of studies. Learning factors were less studied, including engagement with learning (11%), academic progress (9%), general learning skills (4%) and school attendance (2%). When interrogated by age, Table 2 shows that personal/social development and wellbeing has been studied more with secondary aged participants, whereas environmental knowledge and attitudes have been researched more with primary aged children. Academic learning outcomes have been similarly studied across age groups.

**Table 2:** Number of studies by outcome type and age.

	**Infants**	**Primary**	**Secondary**
Self-concept and intrapersonal skills	2	8	40
Social and interpersonal skills	1	11	30
Mental/ emotional health and wellbeing	3	9	15
Environmental knowledge/attitude	1	22	13
Engagement with learning (motivation)		10	12
Academic Progress	1	8	8
General Learning Skills		3	2
School attendance rate	1		2
Other		4	2

*Studies which had multiple outcomes and ages were counted for each category*.

Learning outcomes were also explored across NSLOtC contexts (Table 3). Adventure therapy predictably focused exclusively on intrapersonal and interpersonal development outcomes, and adventure education was similar (although included some measurement of wellbeing and learning engagement). Curricular programs such as field trips, curricular outdoor lessons and school gardens had a mixed focus on both personal development and academic learning outcomes. Residential camps focused more on personal development outcomes, but also included some academic learning outcomes. This likely corresponds with the focus of some camps being curricular (e.g., science camp) and others non-curricular (e.g., summer camp). Unsurprisingly, environmental knowledge and attitude were common outcomes in field trips, residential camps and school gardens, where they were the predictable focus of learning.

**Table 3:** Number of studies by outdoor learning context and measured outcome.

	**Adventure therapy**	**Adventure education**	**Regular field trips**	**Curricular lessons in local outdoors**	**Residential camp**	**School grounds**	**School gardens**	**Other**
Self-concept and intrapersonal skills	4	25		6	5	1	3	3
Social and interpersonal skills	2	12	1	8	11		3	1
Mental/ emotional health and wellbeing		6	2	6	5	1	4	3
Environmental knowledge/attitude		3	7	3	10		7	3
Engagement with learning (motivation)		4	2	6	2		5	2
Academic Progress			3	7	3		4	
General Learning Skills				3	3	1		1
School attendance rate		1	1				1	
Other		1		1			3	

*Studies which had multiple outcomes were counted for each category*.

### Research Quality

Quantitative and qualitative research designs made up 40 and 37% respectively of the total studies, with fewer studies (23%) employing a mixed method design. Forty-four percent of quantitative studies, 35% of mixed methods studies and 2% of qualitative studies utilized a comparison group. Average research quality was rated at a medium level (5.28), with only 50% of quantitative studies and 25% of qualitative studies given a high (7 or above) rating (see Table 4).

**Table 4:** Research quality by study design.

	**Qualitative**	**Quantitative**	**Mixed methods**
Number of studies	54	59	34
Comparison group	1	26	12
Average research quality (−10 to +10)	5.39	5.18	
Standard deviation of research quality	3.57	2.84	
Research quality of 5 or greater	65%	61%	
Research quality of 7 or greater	25%	50%	

*Mixed methods research quality was assessed separately for quantitative and qualitative components*.

When research quality is assessed by outdoor learning context, it can be seen in Table 5 that regular field trips had the lowest quality of research (especially in qualitative studies), along with quantitative research in school gardens. The strongest research quality was in outdoor curricular lessons.

**Table 5:** Average research quality by study design and outdoor learning context.

	**Qualitative**	**Quantitative**
Adventure therapy		5.3
Outdoor adventure education	5.6	5.4
Regular field trips	1.9	3.8
Curricular lessons in local outdoors	6.9	5.7
Residential camp	5.7	5.8
School grounds	4.0	5.5
School gardens	5.7	3.5
Other	5.2	5.1

*Mixed methods research quality was assessed separately for quantitative and qualitative components*.

### Research Outcomes

Research variables were apportioned to broad learning categories as described in Table 2. Almost all studies reported some positive change in the variables they measured; however, in some cases there were mediating factors or other complexities to these results. It is beyond the scope of this narrative synthesis to provide a statistical analysis of outcome effect sizes or to report on the findings of every measured variable. More specific outcomes in each outdoor learning context are outlined in Table 6.

**Table 6:** Common research outcomes by outdoor learning context.

Adventure therapy	Self-concept, coping skills (e.g., resilience, conflict management).
Adventure education	Social and teamwork skills, self-concept, self-esteem, resilience.
Regular field trips	Knowledge about the environment and respect/appreciation for ecosystems.
Curricular lessons in local outdoors	Academic improvement (mixed results), motivation/engagement, wellbeing/mental health, social skills, self-concept.
Residential camp	Connection to nature (mixed results), wellbeing, self-concept and social skills.
School grounds	No common findings.
School gardens	Knowledge of and care for gardens, engagement in learning, social skills and less disruptive behavior.

Further analysis was undertaken of studies with a higher quality rating (five or above), and 52 quantitative studies met this criterion of research quality. Twenty six studies incorporated a comparison or control group, although some compared two types of NSLOtC [e.g., (47)] or different participant groups [e.g., (48)] rather than using indoor classroom learning as the comparison group. The following statistically significant results were noted amongst these higher quality quantitative studies, listed by outdoor learning context:

- Two of the three adventure therapy studies had a mix of positive and negative results, with the third showing significantly increased resilience after the program. These ambivalent outcomes perhaps reflect the challenging population of at-risk youth with complex needs.
- Eighteen adventure education studies all showed significant changes in self-concept and coping factors, specifically including: resilience (7), self-efficacy/self-esteem (4), life effectiveness skills (3) and pro-social behaviors (3). A number of researchers found that the quality of group experience was a moderating factor for these outcomes, and one study (49) interestingly observed that participants with a lower national cultural identification gained less from the adventure education program.
- Three studies of field trips all observed significant improvement in environmental knowledge and attitudes, however none compared these gains with a similar group learning in the classroom.
- Three of the nine studies of curricular lessons in the local outdoors focused on student wellbeing, with two of these noting significant improvement but only one including a control group. Another three studies found curricular lessons in the local outdoors significantly improved motivation and engagement, two of which compared with a control group. Academic progress was recorded in three studies, however the one which included a classroom control group found the same gains. This suggests whilst curricular lessons in the local outdoors may not inhibit or accelerate academic performance, they can particularly impact students' wellbeing and engagement.
- Twelve residential camp studies measured intrapersonal and interpersonal skills, as well as environmental attitudes. Five of these studies found gains in self-concept factors across the program, however only one out of three showed a significant difference compared to a control group. Significant improvement in social skills was noted in six studies of residential camps, three of which included comparison with a control group. Two of four studies measured significant improvement in environmental attitudes, with one of these including a control group. One study (50) found no improvement in environmental attitudes but did show a significant increase in environmentally responsible behaviors. Another study observed that inclement weather during the residential camp actually decreased students' sense of connection to nature (51).
- Two of the three studies of school garden programs found significant improvement in nutritional attitudes and consumption compared to a control group. The third study (52) was a four year primary school gardening program, which found significant gains (compared to a control group) in Mathematics, Languages or Science performance at some time points but not others.

Fifty-three qualitative studies were rated with a research quality of five or higher. Outcome themes of these studies are summarized by outdoor learning context:

- Of the ten adventure education studies, five found improvement in interpersonal skills, and other studies noted benefits to personal factors such as: resilience (2), wellbeing (2), self-concept (2). One study with disengaged high school students (53) found that regular adventure education sessions improved overall school attendance, punctuality and engagement.
- Three studies of regular field trips all focused on deeper environmental awareness, and one study also observed that the open-ended learning context of field trips facilitated students' development of agency, self-regulation and resilience (54).
- In the thirteen studies of curricular lessons in the local outdoors, common outcome themes included: social and teamwork skills (7), wellbeing (5), environmental awareness (4), self-confidence (3) and learning-readiness skills such as concentration and engagement (3). Whilst most studies did not focus on academic achievement, one study of foreign language classes held in the outdoors found that students' fluency improved as well as their confidence and motivation to develop their spoken language skills (55).
- Environmental knowledge and attitudes were the focus of six of the thirteen residential camp studies. Fulfillment of basic psychological needs, as viewed through the lens of Self Determination Theory (56), was regarded as a mediating factor for motivation and wellbeing outcomes in three studies. A deeper understanding of STEM and increased interest in science inquiry was the focus of one residential camp study (57).
- Across the twelve studies of school garden programs, major themes included: self-confidence/self-esteem (6), interpersonal skills (4), wellbeing (3), responsibility (3), independence (3), engagement (3), and environmental attitudes (3). A notable study of high school students with disruptive behaviors (58) found that a gardening program improved school attendance, responsibility for their work, skills development and number of subject pass grades. Two researchers contended that the student-centered pedagogical approach was a mediating factor in the positive outcomes they observed.

## Discussion

This study set out to examine the literature in NSLOtC with the particular purpose of examining the quality of research and the potential for designing school-based learning and development in nature contexts. Depending on the design of the program, NSLOtC can enhance students' health and wellbeing, curriculum and environmental knowledge. An analysis of the literature reveals considerable diversity in terms of types of nature-specific learning offered to school children, but NSLOtC in all its forms affords opportunities for enhanced health and wellbeing, social engagement and curriculum specific outcomes.

Research within each outdoor context has generally been conducted in isolation from others, reducing the global understanding of NSLOtC across various learning contexts. The most commonly studied outdoor learning context was adventure education, which was largely conducted with secondary age students and measured personal and social development outcomes. The lack of long-term follow-up also makes it hard to determine if the outcomes measured impacted on learning across curriculum and years. The other common area of NSLOtC research was in the context of school gardens and curricular lessons, and these focused on primary aged students and explored learning and development factors. Wellbeing benefits were measured less often, however were represented across all outdoor learning contexts. These results might be associated with ease of measurement and the convenience of captive participant groups, rather than the value of the experience *per se*. At the same time, it is worth noting that delivery of each outdoor learning experience requires different skillsets.

The quality of NSLOtC research varied greatly, as has been noted in previous similar reviews (3, 19, 33). This could reflect the importance of the learning environment in facilitating the measured responses, especially considering the impact of activities in natural environments on data collection. Research examining regular field trips was of particularly poor quality, most likely reflecting the potential confounding variables of this type of experience. Most NSLOtC studies had at least moderate quality, and curricular lessons in the local outdoors had the highest rigor across quantitative and qualitative research designs.

Interpreting the value of NSLOtC beyond the participant group being studied is constrained by participant cohorts and cultural perspectives. As a significant number of studies were conducted in North America and the United Kingdom, the cultural impact of NSLOtC design and relevance of outcomes measured is unclear. Research across different cultures, and that specifically considers diversity and inclusion within student groups, will enrich our understanding of how best to design NSLOtC. While there is a small research base documenting wellbeing and mental health benefits of outdoor learning, these outcomes need to be studied more thoroughly across age groups and learning contexts. Outdoor curricular lessons and learning in school gardens have clear benefits for primary school aged students, and these also need to be studied in secondary settings. Conversely, the personal and social gains secondary students experience from adventure education should be further researched for primary students. Pro-environmental knowledge and attitude have mainly been studied in the contexts of residential camps, field trips and school gardens; however, they also need to be studied in adventure and curricular outdoor learning contexts to explore whether improvements can be achieved in those settings also.

There are a number of studies showing neutral or positive impacts for NSLOtC on academic outcomes for both primary and secondary students, compared with control groups. This finding is intriguing as it suggests that the indoor classroom may not be the only environment where effective academic learning occurs, not to mention the socio-emotional learning opportunities provided by natural outdoor settings. While this needs to be researched more thoroughly, the implications are that classroom teachers might benefit from initial teacher education curricula and professional development that enables them to deliver learning in nature-specific environments. Many of the NSLOtC studies described a student-centered style of learning, and a different student relationship with adult learning facilitators compared to a regular classroom teacher. These learning-mode factors could mediate the effectiveness of NSLOtC, and research comparing outdoor and indoor learning using similar teaching and learning approaches would help to tease out causal factors. Future research could include studies examining the impact of different environments, as well the relationship between environment and teaching and learning design. One size does not fit all, so in the first instance research design will need to reflect this contextual approach.

The current body of research has established that various manifestations of NSLOtC can effectively produce holistic learning, wellbeing and development outcomes; however, comparison across outdoor learning contexts has been rarely studied. Miller et al. (33) noted that it is currently unclear which elements of nature-based learning positively impact engagement and social outcomes. The next challenge for NSLOtC research is to explore the mechanisms of this demonstrated effectiveness, in order to uncover foundational elements which are common across outdoor learning settings, and other factors which are context-specific. In calling for a unification in NSLOtC research, however, we caution against a “drag and drop” approach to NSLOtC practice, where an effective model in one context is replicated in another setting without regard to local educational, environmental and cultural factors (59).

In order to achieve a high level of rigor, future NSLOtC research should incorporate the following elements:

- Thorough description of the NSLOtC intervention including: participant background, contextual factors, frequency and duration of sessions, pedagogical approach, students' experience of the intervention, and justification for chosen measures.
- A comparison or control group learning the same material in an indoor classroom setting or an alternate outdoor setting, to ascertain whether the chosen type of NSLOtC has any additional benefit to the learning variables of interest.
- A mixed methods and/or longitudinal design, including quantitative measures of objective change and/or in-depth qualitative approaches.
- Conclusions which go beyond simply *whether* NSLOtC is effective, and rather present a more sophisticated picture of *how* it works most effectively for *whom* and in *what* settings.

This review builds on evidence from previous systematic reviews which assessed research in natural environments (30–32). In general, those reviews reflect the findings in the current systematic review, where nature-based experiences enhance mental health, emotional regulation and environmental knowledge and attitudes. Systematic reviews that focused on educational experiences in natural settings (3, 19) similarly found social and academic benefits. While previous reviews did not focus on school-aged children alone or educational learning specifically, the current review adds to and consolidates this evidence base by reviewing natural outdoor learning experiences across all school aged children, and incorporating curricular and non-curricular learning contexts.

Although the large scale of this review suggests findings are indicative of widespread benefits, the current systematic review had some limitations. Gray literature was not accessed due to the extensive size of the 16,000 items in the initial dataset, and although articles were double screened for inclusion, their research quality was only rated by a single reviewer for each paper. Further, some databases (e.g., SPORTDiscus), which may have yielded further NSLOtC research, were not accessed. One criterion of inclusion was that articles were written in English, which would have excluded a small number of studies published in other languages and skewed the reported international spread of research and culturally specific interpretations. Even within English speaking countries, there are a wide variety of cultural and pedagogical contexts within which NSLOtC occurs, and therefore the general findings of this review should not be applied to individual contexts without due regard to local factors. The most significant limitation of the current review was that a statistical analysis of outcome effects was not possible due to the variety of study designs and broad range of measured outcomes.

There is sufficient research evidence to show the importance of NSLOtC experiences for school students. The following recommendations are made by the international team which conducted this systematic review:

1. Educational policy-makers need to recognize that NSLOtC is no longer a fringe “nice-to-have” approach to teaching and learning. Instead, NSLOtC is an effective pedagogical approach for holistic growth, which means that resources should be made available for implementation in curricula and learning design [(60), in press].
2. All university pre-service teacher programs should include skill development activities for the design and implementation of NSLOtC in the upcoming teacher's subject area.
3. Design and implementation of NSLOtC is an important part of current practicing teachers' skill development, and professional development courses should be designed and delivered for in-service teachers.
4. Future research in NSLOtC needs to be carefully designed following rigorous methodologies to evaluate and differentiate between nature-specific and classroom settings, various outcomes, and what elements contribute to these outcomes.
5. Future researchers might also focus on designing effective NSLOtC research studies, which lead to rigorous findings.
6. Environments which are local to, and within, school grounds need to provide opportunities for everyday NSLOtC experiences.

## Conclusion

This systematic review built upon the work of previous research reviews by incorporating wellbeing, socio-emotional development and academic learning outcomes for school aged children in various nature-based outdoor learning contexts. The 147 included studies indicated significant support for the benefits of NSLOtC, particularly in personal and social development for secondary aged students in adventure education programs, and in social and academic learning outcomes for primary aged students in school garden and curricular outdoor learning settings. The review identified that most NSLOtC research has been conducted in North America and the United Kingdom, and that research in each outdoor learning context tended to be clustered with specific outcome types and student age groups. The quality of research was moderate on average, although there was a wide range. Recommendations from this systematic review include: the provision of NSLOtC in national and state curricula, subject-specific training in NSLOtC for pre-service and in-service teachers, and rigorous design of future NSLOtC research.

## Data Availability Statement

The original contributions presented in the study are included in the article/supplementary material, further inquiries can be directed to the corresponding author/s.

## Author Contributions

All authors listed have made a substantial, direct, and intellectual contribution to the work and approved it for publication.

## Conflict of Interest

The authors declare that the research was conducted in the absence of any commercial or financial relationships that could be construed as a potential conflict of interest.

## Publisher's Note

All claims expressed in this article are solely those of the authors and do not necessarily represent those of their affiliated organizations, or those of the publisher, the editors and the reviewers. Any product that may be evaluated in this article, or claim that may be made by its manufacturer, is not guaranteed or endorsed by the publisher.
